# The transition from school to university in mathematics education research: new trends and ideas from a systematic literature review

**DOI:** 10.1007/s10649-022-10194-w

**Published:** 2022-11-11

**Authors:** Pietro Di Martino, Francesca Gregorio, Paola Iannone

**Affiliations:** 1 Department of Mathematics, Università Di Pisa, Largo B. Pontecorvo 5, 56125 Pisa, Italy; 2 Université de Paris, Univ. Paris Est Creteil, CY Cergy Paris Université, Univ. Lille, UNIROUEN, LDAR, F-75013 Paris, France; 3 Haute École Pédagogique du Canton de Vaud, Lausanne, Switzerland; 4 Department of Mathematics Education, Loughborough University, Loughborough, LE11 3TU UK

**Keywords:** Secondary-tertiary transition, Systematic literature review, Epistemological affective and socio-cultural approaches, Undergraduate mathematics students, Rite of passage

## Abstract

Investigating the transition between educational levels is one of the main themes for the future of mathematics education. In particular, the transition from secondary school to STEM degrees is problematic for the widespread students’ difficulties and significant for the implications that it has on students’ futures. Knowing and understanding the past is key to imagine the future of a research field. For this reason, this paper reports a systematic review of the literature on the secondary-tertiary transition in Mathematics Education from 2008 to 2021. We constructed two corpuses: one from the proceedings of three international conferences in mathematics education (PME, ICME, and INDRUM) and the other from peer reviewed research papers and book chapters returned by the databases ERIC and Google Scholar. A clear evolution in perspectives since 2008 emerges from the analysis of the two corpuses: the research focus changed from a purely cognitive to a more holistic one, including socio-cultural and — to a lesser extent — affective issues. To this end, a variety of research methods were used, and specific theoretical models were developed in the considered papers. The analysis also highlights a worrisome trend of underrepresentation: very little research comes from large geographical areas such as South America or Africa. We argue that this gap in representation is problematic as research on secondary tertiary transition concerns also consideration of socio-cultural and contextual factors.

## Introduction

The study of the transition from secondary school to university mathematics — also referred to as the secondary-tertiary transition (STT) — has a long tradition in mathematics education research. Seminal studies were conducted by Tall since the 1980s, investigating significant cognitive discontinuities in STT (Tall, 1991; Tall & Vinner, 1981). Tall’s studies inspired the development of research specifically focused on students’ difficulties in STT. At the end of the past millennium, De Guzman et al. (1998) summarised the main findings of this field of research and identified three categories related to students’ difficulties in STT: epistemological-cognitive, sociological-cultural, and didactical.

A quarter of a century later, students’ difficulties in STT are still a significant issue for modern society. The high dropout of undergraduate students in STEM subjects reported in several countries (Rach & Heinze, 2017) is problematic for at least two reasons. The first relates to the need of advanced mathematics competencies for economies to flourish (Adkins & Noyes, 2016). The second is linked to equity, given the opportunities afforded by STEM degrees (science, technology, engineering, and mathematics) in terms of social mobility and future earnings (see Higher Education Statistics Agency, 2018, for the UK context). Indeed, the European Mathematical Society recently conducted a survey amongst mathematicians to collect information to devise national and international actions that may help reduce students’ dropout during the STT (Koichu & Pinto, 2019). The authors underline that the STT is a complex and multi-faceted process, provoking frustration in first-year students, as well as in lecturers.

The difficulties that students encounter when moving from school mathematics to the mathematics in a STEM degree still represent a strange story, not fully explained, that involves and overwhelms even students considered excellent in mathematics during their school experience (Di Martino & Gregorio, 2019). Understanding this complex and apparently unexplained phenomenon requires many theoretical approaches and, as Schoenfeld (2000) notices, ‘findings are rarely definitive; they are usually suggestive. Evidence is not on the order of proof but is cumulative’ (p. 648). This cumulative nature of findings suggests the relevance of literature reviews on selected topics: they allow researchers to identify trends and provide insights for possible developments of the research field (Pan, 2016).

An influential review of research on STT was conducted by Gueudet in 2008. Gueudet highlights three types of transitions involving individual, social, and institutional factors: transition in ways of thinking, transition to proof and the technical language of mathematics, and institutional transition related to changes in the didactical contract. Gueudet’s review also confirms that up to that time, the tertiary transition in mathematics was mainly studied through cognitive and epistemological lenses, even though socio-cultural and affective issues had since assumed an important role in mathematics education (Lerman, 2000). Gueudet (2008) concludes that there is ‘the need for further research, and for teaching designs grounded in their findings’ (p. 252). Her call was heard by the mathematics education research community, and research on STT has since adopted new theoretical perspectives, discussed new results, and highlighted new lines of interest. Indeed, the transitions to higher education are one of the themes emerging from a recent international survey of researchers in mathematics education answering the question: ‘on what themes should research in mathematics education focus in the coming decade?’ (Bakker et al., 2021, p. 2).

Recently, Bergsten and Jablonka (2019) traced the development of studies on STT in terms of the theoretical approaches taken. However, a systematic review of the research on STT in the last 15 years is still missing. To fill this gap, we analyse the research on STT in mathematics education since 2008.

We developed our systematic review to answer the following research questions related to the period 2008–2021:

RQ1: Which are the methods and the specific theoretical frameworks used to approach STT?

RQ2: What are the main themes of STT research emerging from an analysis of published research?

RQ3: What is the geographical distribution of STT research? Do we know enough about diverse cultural context coming from a wide range of countries?

## Methodology

The development of a systematic review involves several aspects: in this section, we briefly describe the underlying choices of our review and their reasons, as well as its constraints and the possibility of reproducibility.

The development of a literature review is a systematic process (Pan, 2016), involving three steps needed to identify the corpus of interest (Green et al., 2006): selection of the topic, definition of the sources of information, and definition of the selection criteria employed (Table 1).

**Table 1:** The three steps for a literature review (Green et al., 2006)

Step 1: Selection of the topic	Step 2: Sources of information	Step 3: Selection criteria
Why the topic is relevant?	Database	Automatic search criteria
Why a literature review about the topic is needed?	Starting and ending year of the search	Selection criteria used to include or exclude in the review a study selected by the automatic criteria

The relevance of the topic and the reasons for the review (Step 1) were discussed in the introduction of this paper.

The description of Steps 2 and 3 is of crucial importance for a systematic literature review: it allows other scholars to replicate the selection of the corpus by following the given criteria. Related to these two steps, we differentiate between the two searches we conducted: one focused on conference proceedings, the other on refereed journal papers and book chapters. For both searches, the starting year was 2008 and the ending was October 2021. However, the database and, in part, the automatic search criteria were different between the two searches.

According to our focus, we included in this review studies describing the STT to university mathematics of students enrolled in STEM degrees. These studies may involve pre-service secondary mathematics teachers in those countries where they are taught mathematics in the same lectures of students enrolled in a STEM degree. However, we consider the STT for pre-service teachers (including teachers for primary and lower secondary education) as a special case of transition to tertiary mathematics deserving, in our view, a separate discussion, not addressed in this paper.

We developed a specific search for conference proceedings since international conferences are likely to be a forum to share ideas which are then developed in journal papers or book chapters, and results emerging from conference proceedings often anticipate trends in the development of research themes.

The first choice we made was related to which conference’s proceedings to include in the review. We decided to include two of the most popular (by number of participants) international conferences in mathematics education: the International Congress on Mathematical Education (ICME) and the Conference of the International Group for the Psychology of Mathematics Education (PME). For the likely relevance to our focus, we also included the conferences organised by the International Network for Didactic Research in University Mathematics (INDRUM).

Since our third research question concerned the representation of geographical regions in mathematics education research, one of the main reasons for the selection of ICME, PME, and INDRUM was that the international program committees of these conferences are opened to members coming from all over the world. Using this criterion, we did not include in our search other potentially relevant conferences such as CERME (Congress of the European society for Research in Mathematics Education), RUME (the Research on Undergraduate Mathematics Education of the Mathematical Association of America), PME-NA (North American Chapter of the International Group for the Psychology of Mathematics Education), and other PME regional conferences.

The database for the construction of this first corpus therefore consisted of the published proceedings of ICME, PME, and INDRUM since 2008. We identified all the contributions including the key term ‘transition’ in the title, abstract, or text (automatic search criterion). We then considered only contributions where the occurrence was related to the secondary-tertiary transition and with STT as the significant focus of the paper (not mentioned as a secondary topic).

For the construction of the second corpus (which includes journal papers and book chapters), we first considered what search criteria to use, as this is a crucial step for every literature review. Gusenbauer and Haddaway (2020) recognised three main quality criteria for literature searches: completeness (identifying all the most significant resources about the topic), transparency, and reproducibility. Through the analysis of the systematic search qualities of 28 academic search engines, the authors suggested to conduct literature reviews using at least two different search engines, according to their quality with respect the three criteria mentioned above. Regarding Google Scholar, Gusenbauer and Haddaway (2020) observe that this search engine has several limitations in terms of transparency and reproducibility of searches; however, ‘it is considered a suitable supplementary source of evidence (including on grey literature)’ (p. 196) for systematic literature reviews.

Based on the above considerations, we used two databases: ERIC (Education Resources Information Center, https://eric.ed.gov), the online database of education research promoted by the US Department of Education, and Google Scholar (https://scholar.google.com). The use of Google Scholar in our review appeared important to bring to the fore contributions by researchers who may not have access to the main journals and conferences of our community (for example due to the high cost of conference fees and travel). We also followed Haddaway et al.’s (2015) advice to focus on the first 200 to 300 results returned by Google Scholar for systematic reviews as supplementary source of evidence.

Since the databases were not specific to mathematics education, we used a strict automatic search criterion. We used ‘transition AND mathematics AND school AND university’ as search terms. We observed a posteriori that the inclusion of the word ‘university’ in the Google Scholar search was not useful: Google Scholar searches the entire document — unlike ERIC which searches only the title, the abstract, and the keywords — and therefore the word university featured in all documents where the names of authors and their institutions were included. At the end of this first phase, following the recommendation of Haddaway et al. (2015), we considered the corpus produced by the union of the 252 results produced from ERIC and the first 300 results by relevance from Google Scholar taking care to eliminate repetition of entries between the two sets of results since, as expected, there was a significant overlap.

The selection criteria used to include a study identified using the automatic search criteria were the same for both databases. Assuming the definition of STT given in the Encyclopaedia of Mathematics Education as ‘the process experienced by students leaving secondary school and entering different kinds of postsecondary institutions: universities, engineering schools, etc.’ (Gueudet & Thomas, 2020, p. 762), we selected the contributions describing a transitional process*,* considering a ‘before’ and an ‘after’ this process. This means we excluded studies where the focus was solely on one of the stages of this transition (e.g., students’ experiences with proof at the start of a mathematics degree) and did not consider the ‘before’ and ‘after’ of the STT. Because of this choice, papers focusing on the design of transition to proof courses or university preparatory classes were not included in our review.

A margin of subjectivity in the application of the selection criteria clearly exists in this process. However, the first two authors developed an investigator triangulation to balance individual biases (Mok & Clarke, 2015). The two authors applied independently the selection criteria, sharing their outcome. They then discussed the cases of disagreement until a full agreement for the selection of the final corpuses was reached.

The result of this process was the two final corpuses: the first one including 59 reports presented in the three selected conferences and the second one including 55 papers from peer reviewed journals and books.

Once we selected the corpus, aligned with the research questions of the systematic review, each paper was categorised according to: year of publication, type of study (theoretical, empirical, didactical design) and — if empirical — methods (quantitative, qualitative, mixed), instruments, sample, context^1^ (country where the research was developed, how many and which schools, university, or texts were involved in the research), theoretical framework(s), research question(s), and focus (cognitive, affective, socio-cultural). This classification allowed for multiple labels: for example, a paper could be classified with cognitive and socio-cultural foci if explicit references to both cognitive and socio-cultural issues were made in the theoretical framework or in the research question(s).

In addition to the described classification, we also noted the main findings of the study for each paper.

**Fig. 1:**
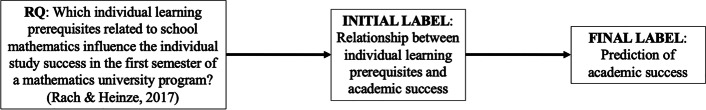
Example of the coding process for the research questions

The recognition of the main research themes was based on a coding process related to the research questions aimed to recognise frequencies and patterns (Cohen et al., 2007). Initially, a very specific label was assigned to each research question of the paper considered, then — following Miles et al. (2003) — the labels evolved during the process to describe wider categories capable to bring together several similar focuses (an example of this evolution is summarised in Fig. 1).

## Results and discussion

### STT in conference proceedings from 2008 to 2021

As previously described, we considered in our review three international conferences: ICME, INDRUM, and PME.

#### ICME congresses

ICME is a large international congress held every 4 years involving researchers worldwide. Four ICME congresses took place since 2008: the proceedings of ICME-11 (Monterrey, Mexico 2008) and of ICME-14 (Shanghai, China, 2021, online conference) are not currently available, while those of ICME-12 (Seoul, Korea, 2012) and ICME-13 (Hamburg, Germany, 2016) are published as open access Springer books. According to the spirit and nature of the activities of the Topic Study Groups (TSG), the existing proceedings consist of abstracts of the discussions developed in the sessions rather than a collection of research reports. For this reason, the discussion which follows is short and mainly descriptive.

The evolution of the presence of STT in the discussion of the ICME community can be recognised by the analysis of the proceedings, scientific programs, and books related to developed activities. In what follows, we detail the evolution of research on STT within these four ICME congresses.

Although one of the TSGs of ICME-11 was about university mathematics, STT was not mentioned in the description of its scientific program.

The situation was different 4 years later at ICME-12. The transition ‘problem’ was explicitly mentioned in the call for papers and in the description of TSG 2: ‘Mathematics education at the tertiary level and access to tertiary level’. Within this TSG, we found five contributions on STT by authors from five different continents (Brazil, Canada, South Korea, South Africa, Sweden). Four out of these five contributions analysed students’ difficulties with specific mathematics topic (matrices, axiomatic method, calculus) with a predominantly cognitive approach. There was also a survey team dedicated to STT, and the outcomes of this work were findings of an international survey of 79 mathematics lecturers from 21 countries. Findings pointed to ‘a multi-faceted web of cognitive, curricular and pedagogical issues’ (Thomas et al., 2015, p. 278) related to STT, also depending on the institutional context. Despite the evident differences between institutions, and therefore the emergence of socio-cultural aspects, the lens of this survey is still strongly cognitive, without specific reference to affective issue. However, the final mention of the emerging interest for how students experience their first encounters with advanced mathematical topics represented a first step towards the inclusion of affective issues in the discussion.

At ICME-13, STT was a recurrent topic: seven contributions presented in TSG2: ‘Mathematics education at the tertiary level’ were focused on STT. In addition, transition issues were the topic of a discussion group and of the plenary panel. The latter inspired an ICME monograph where the main results of the research and ‘the blind spots that remain unquestioned’ (Gueudet et al., 2016, p. 19) were discussed. The summary of the results included considerations about institutional differences (for example in class size or equipment) and pedagogical and cognitive issues, but no mention of affect was made.

At ICME-14, four contributions in TSG2 were focused on STT. In particular, Pinto, Gamlieli, and Koichu discussed the results of an international survey conducted among 310 tertiary mathematics lecturers from 30 countries. This confirms the potential of ICME Congresses to foster discussion between researchers from different contexts. The wide geographical reach of the ICME Congresses is particularly relevant for research on STT considering that international comparisons are still rare elsewhere in our field.

To conclude, the diachronic analysis of the proceedings and scientific programmes of these four ICME editions shows an increasing interest for STT. However, while a lack of attention to affective issues clearly emerges, the discussion about socio-cultural issues is promoted through the involvement of researchers from a variety of countries, with a considerable participation of researchers from countries currently underrepresented in mathematics education.

#### INDRUM conferences

The growing interest for undergraduate mathematics education in our field is also evidenced by the foundation of the International Network for Didactic Research in University Mathematics (INDRUM) in 2015. Since 2016, this network holds a biannual conference with open access to online proceedings. To date, the following conferences have been held: INDRUM 2016 (Montpellier, France, Nardi et al., 2016), INDRUM 2018 (Kristiansand, Norway, Durand-Guerrier et al., 2018), and INDRUM 2020 (virtually held in Bizerte, Tunisia, Hausberger et al., 2020). The book *Research and Development in University Mathematics Education* (Durand-Guerrier et al., 2021) provides a detailed overview of the discussion on topics in the 2016 and 2018 INDRUM conferences. Focusing on STT and according to our criteria, we identified 18 contributions relevant to our review (Table 2).

**Table 2:** INDRUM proceedings: the complete list of selected publications

Author(s)	Type of study	Method	Sample and instruments	Context
Artigue (2016)	Theoretical	–	–	France
Bloch and Gibel (2016)	Empirical	Qualitative	14 university students (students’ scripts from the final exam)	France
Gueudet and Pepin (2016)	Empirical	Qualitative	86 university students, 1 university teacher (interview, online questionnaire, students’ scripts)	France and UK
Hausberger (2016)	Theoretical	–	–	France
Nihoul (2016)	Empirical	Qualitative	24 university students (students’ scripts)	Belgium
Petropoulou et al. (2016)	Empirical	Qualitative	1 lecturer (direct observation of lessons, interview)	Greece
Quéré (2016)	Empirical	Qualitative	179 university students (online questionnaire)	France
Vandebrouck and Leidwanger (2016)	Empirical	Mixed	513 university students (online questionnaire)	France
Brandes and Hardy (2018)	Theoretical	–	–	Canada
Broley and Hardy (2018)	Empirical	Qualitative	Analysis of lecturers’ assignments and exams	Canada
Geisler and Rolka (2018a)	Empirical	Quantitative	193 university students (questionnaire)	Germany
Gradwohl and Eichler (2018)	Empirical	Quantitative	182 university students (questionnaire)	Germany
Hochmuth (2018)	Theoretical			Germany
Kock and Pepin (2018)	Empirical	Qualitative	24 university students, 2 lecturers, 1 tutor (interview)	The Netherland
Rach et al. (2018)	Empirical	Quantitative	339 university students (questionnaire, students’ script)	Germany
Doukhan (2020)	Empirical	Quantitative	29 secondary students, 25 university students (students’ scripts)	France
Flores Gonzalez et al. (2020)	Empirical	Qualitative	1 lecturer (Analysis of course reference books, interview)	Brazil
Khalloufi-Mouha (2020)	Empirical	Qualitative	1 lecturer (Analysis of two schoolbooks and lecturer’s notes)	Tunisia

We first notice a clear prevalence (78%) of contributions by European researchers, probably related to the origin of the INDRUM group. Within this European prevalence, there is a strong presence of French authors who account for 39% of the reports on STT. The French influence on the INDRUM community is not only evident in terms of participation: it is also evident from the theoretical frameworks used in the research reports included in the proceedings of these conferences.

Three out of the four theoretical papers on STT used the anthropological theory of the didactic (ADT) (Chevallard, 1992) for identifying praxeologies in STT. The fourth, the paper by Artigue (2016), discussed the challenges of the research in mathematics education at the tertiary level. As for the empirical papers, four reported and interpreted students’ difficulties in STT using the lens of the anthropological theory of didactic. The common hypothesis of these studies was that several phenomena in STT can be interpreted as institutional issues, determined by the strong discontinuity between school and university praxeologies. This approach was extensively described in Gueudet and Pepin (2017).

Using this framework, Winsløw (2008) introduced a model with repeated cycles of two transitions to university mathematical praxeologies: the first is related to the need for students to extend their praxeologies considering the role of theory in mathematics. The second transition takes place when the elements of the theory become objects; students need to work with autonomously: in this case, the emergence of new objects can require further transitions.

Other important theoretical frameworks developed by French researchers and adopted in papers included in the INDRUM proceedings for studying STT are the theory of didactical situations (Brousseau, 2002) — that Bloch and Gibel (2016) used for developing a tool for modelling students’ reasoning processes — and the Instrumental Approach (Rabardel, 2002), used by Gueudet and Pepin (2016) for theorising the use of technology in STT.

The INDRUM papers pay great attention to cognitive and epistemological issues in STT; several of the authors’ theoretical approaches fall ‘under the umbrella term advanced mathematical thinking’ (Hochmuth et al., 2021, p. 195) and are often situated within APOS (action-process-object-schema) theory (Dubinsky, 1991). However, the use of the ATD also shows some consideration of institutional and social perspectives, but only one paper (Gueudet & Pepin, 2016) analysed and compared two case studies from different national contexts (France and UK).

Only two papers in our INDRUM sample have an affective focus. Quéré (2016) analysed the different forms of autonomy required in the move from secondary to tertiary education in mathematics, also discussing the role of theories of success and expectations in students’ difficulties in STT. Geisler and Rolka (2018b) discussed the relationship between students’ success in mathematics in their first university year and some affective variables (self-concept, interest, view of mathematics, basic needs, self-efficacy).

#### PME conferences

PME conferences have annual frequency; therefore, we analysed 13 editions: from Mexico 2008 to the edition virtually held in Thailand in 2021.^2^ PME includes several types of contributions and activities: research report, short oral, working group, discussion group, poster, and plenary. In the following, we will focus on the 26 selected research reports (Table 3).

**Table 3:** PME proceedings: the complete list of selected publications

Author(s)	Type of study	Method	Sample and instruments	Context
Dahl (2009)	Empirical	Qualitative	Analysis of official curricula	Denmark
Di Martino and Maracci (2009)	Didactical Design	–	–	Italy
Klymchuk and Thomas (2009)	Empirical	Qualitative	178 school and university teachers (questionnaire)	New Zealand
Dias et al. (2010)	Empirical	Qualitative	Analysis of official curricula and tasks used for the entrance selection	Brazil, France
Rach and Heinze (2011)	Empirical	Quantitative	118 university students (questionnaire, final school grades)	Germany
Halverscheid and Pustelnik (2013)	Empirical	Quantitative	294 university students (exam results)	Germany
Hong and Thomas (2013)	Didactical Design	–	–	Korea, New Zealand
Schäfer (2013)	Empirical	Qualitative	2 pairs of students (interview)	Germany
Beitlich et al. (2014)	Empirical	Quantitative	6 lecturers and 2 two university students (eye tracking)	Germany
Halverscheid et al. (2015)	Empirical	Quantitative	1134 university students (test results)	Germany
Ufer (2015)	Empirical	Quantitative	333 university students (students' scripts from a text, final school grades, self-report about study motives)	Germany
Jeschke et al. (2016)	Empirical	Quantitative	661 university students (questionnaire, measures of self-concept and interest in mathematics)	Germany
Ottinger et al. (2016)	Empirical	Quantitative	159 university students (students’ scripts)	Germany
Pustelnik and Halverscheid (2016)	Empirical	Quantitative	584 university students (test results, degree course, time gap, and duration of school attendance)	Germany
Griese (2017)	Empirical	Quantitative	255 university students (questionnaire)	Germany
Kouvela et al. (2017)	Empirical	Qualitative	2 students (interview, focus group)	UK
Pigge et al. (2017)	Empirical	Quantitative	36 university teachers (online questionnaire)	Germany
Bampili et al. (2018)	Empirical	Qualitative	2 university students (interview)	Greece
Di Martino and Gregorio (2018)	Empirical	Qualitative	127 university students (questionnaire, interview)	Italy
Geisler and Rolka (2018b)	Empirical	Quantitative	155 university students (2 questionnaires)	Germany
Maciejewski (2018)	Empirical	Mixed	116 university students (short answers to an open prompt)	USA
Meehan et al. (2018)	Empirical	Quantitative	33 university students (questionnaire)	Ireland
Geisler and Rach (2019)	Empirical	Quantitative	89 mathematics freshmen (2 questionnaires)	Germany
Roos (2019)	Empirical	Mixed	89 university students (questionnaire)	Germany
Geisler (2021)	Empirical	Quantitative	222 university students (two questionnaire)	Germany
Rach (2021)	Empirical	Quantitative	150 university students (questionnaire)	Germany

Table 3 illustrates both a small but steadily growing presence of empirical research reports on STT since 2009, and the absence of theoretical reports. The latter may also result from the strict length limitation of the contributions. The analysis of representation by country returns a clear picture: 23 out of 26 reports (88%) were developed in a European country by European researchers (one of these papers discusses a comparison between France and Brazil, also involving a Brazilian author). These data highlight an issue of underrepresentation in the PME conferences concerning the discussion about STT.

Moreover, 16 (62%) out of 26 reports are by researchers from German universities. This regional predominance is also reflected in the research methodologies: the quantitative approach is prevalent in these empirical studies (62.5% versus 29% for the qualitative approach and 8% for the mixed one), and samples are usually large, notwithstanding the presence of three interesting case studies. Several reports focus on the identification of significant correlations between academic success/failure and other variables. This aim is related to a clear definition of the variables involved, to the development of instruments for measuring these variables and the students’ success or failure, and to the adoption of statistical models (the more frequently adopted being the Rasch model).

Considering the 7-year period 2008–2014, we found 9 research reports about STT measuring students’ preparedness in mathematics and students’ learning strategies at the beginning of their university experience. These studies aim to determine whether and to what extent mathematics dropout can be predicted by the analysis of cognitive performance at the beginning of university (Halverscheid & Pustelnik, 2013). In first period, two exceptions are represented by the paper by Di Martino and Maracci (2009), stressing the need to go beyond a purely cognitive approach also in research about STT, and the paper by Dias et al. (2010), that, within the ATD framework, developed a socio-cultural comparison between Brazil and France regarding STT, considering differences in educational systems and in educational cultures.

In the subsequent 7-year period (2015–2021), we found 17 research reports about STT. The clear difference in the number of reports between the period 2008–2014 and the period 2015–2021 highlights the growing interest for STT in the mathematics education community. The analysis of the frameworks and the research questions used in the research reports in the period 2015–2021 shows a greater consideration for affective and socio-cultural constructs in STT research. Ufer (2015) analysed the relationship between students’ motivations to choose a mathematical programme and their success, Jeschke et al. (2016) attempted to measure students’ ‘academic buoyancy’ and its role in early dropout, and Kouvela et al. (2017) studied the students’ identities as mathematics learners and the influence that messages given by their lecturers have on the development of these identities.

The 2018 edition of PME in Umeå deserves special attention because of its variety of approaches to STT. Bampili et al. (2018) analysed how social and institutional issues shape the development of a new identity for first year mathematics students by adopting the theoretical framework of Communities of Practice (Wenger, 1998). Meehan et al. (2018) studied how STT affects high-achieving students’ ‘sense of belonging to math’, that is related ‘to whether one feels a member of a mathematical community and feels valued and accepted by that community’ (p. 371). Di Martino and Gregorio (2018) introduced and analysed the so called ‘first-time phenomenon’, that is the cognitive and emotional reactions of (successful) students to the first experience of failure in mathematics. Geisler and Rolka (2018b) discussed the relationship between affective variables (such as self-concept and beliefs about the nature of mathematics) and academic procrastination.

Therefore, the diachronic analysis of the research reports on STT in the 13 PME conferences shows a clear evolution toward a more holistic view of STT that increasingly includes socio-cultural and affective considerations.

### STT in journal papers and book chapters

Following the selection criteria described in the method section, we obtained a corpus of 55 papers (Table 4). As hypothesised, we found several papers extending the ideas discussed by the authors in the international conferences presented in the previous section; therefore, the analysis of this corpus is particularly significant to gain a clear picture of the state of the art of STT research.

**Table 4:** Journals and books: the complete list of selected publications

Author(s)	Type of study	Method	Sample and instruments	Context
Brandell et al. (2008)	Empirical	Qualitative	Analysis of official curricula	Sweden
Clark and Lovric (2008)	Theoretical	–	–	Australia, Canada
Godfrey and Thomas (2008)	Empirical	Mixed	Three groups of students: in the lower secondary school, upper secondary school, and first year at university	New Zealand
Tall (2008)	Theoretical	–	–	UK
Clark and Lovric (2009)	Theoretical	–	–	Australia, Canada
Hong et al. (2009)	Empirical	Quantitative	178 schoolteachers, 26 lecturers (questionnaire, interview) in New Zealand	Thailand, New Zealand
Liston and O’Donoghue (2009)	Empirical	Quantitative	607 university students (questionnaire)	Ireland
Culpepper et al. (2010)	Empirical	Quantitative	2.108 students (results in standardised test)	USA
Engelbrecht (2010)	Theoretical	–	–	South Africa
Liston and O’Donoghue (2010)	Empirical	Qualitative	15 university students (semi-structured interviews)	Ireland
Hernandez-Martinez et al. (2011)	Empirical	Qualitative	School teachers and managers (interview and observation of lessons)	UK
Klymchuk et al. (2011)	Empirical	Mixed	63 lecturers from 24 countries (questionnaire)	New Zealand, Germany, Australia
Lithner (2011)	Theoretical	–	–	Sweden
Bardelle and Di Martino (2012)	Didactical Design	–	–	Italy
Pampaka et al. (2012)	Empirical	Quantitative	University students (questionnaire)	UK
Selden (2012)	Theoretical	–	–	USA
Thomas and Klymchuk (2012)	Empirical	Mixed	178 schoolteachers, 26 lecturers (questionnaire, interview, observation of lessons)	New Zealand
Varsavsky (2012)	Empirical	Quantitative	1240 university students (students’ academic results)	Australia
Adamuti-Trache et al. (2013)	Empirical	Quantitative	4.569 school students in USA over the 5-year period 2002–2006 (student gender, graduation year, high-school attended, performance in first year)	Canada, USA
Daza et al. (2013)	Didactical Design	–	–	Spain
Gueudet (2013)	Theoretical	–	–	France
Hernandez-Martinez and Williams (2013)	Empirical	Qualitative	2 university students (narratives)	UK
Duah et al. (2014)	Empirical	Mixed	Observation of 8 peer-assisted learning sessions	UK
Pepin (2014a)	Empirical	Mixed	1 university student in UK (interview with student and his teachers/lecturers, documents from the school and the university of the student)	Norway
Pepin (2014b)	Empirical	Qualitative	1 university student in UK (interview with student and his teachers/lecturers, documents from the school and the university of the student)	Norway
Vollstedt et al. (2014)	Theoretical	–	–	Germany
Hong and Thomas (2015)	Empirical	Mixed	136 university students in Korea (students’ scripts)	Korea, New Zealand
Hernandez-Martinez (2016)	Empirical	Qualitative	2 university students (narratives)	UK
Jooganah and Williams (2016)	Empirical	Qualitative	8 university students, 12 lecturers (interviews)	UK
Bengmark et al. (2017)	Empirical	Quantitative	361 university students (2 questionnaires)	Sweden
Corriveau (2017)	Empirical	Qualitative	3 schoolteachers, 3 lecturers (teachers’ scripts)	Canada
Corriveau and Bednarz (2017)	Empirical	Qualitative	3 schoolteachers, 3 lecturers (verbatim transcriptions of the meetings: 36 h)	Canada
Jablonka et al. (2017)	Empirical	Qualitative	60 university students in Sweden (interview)	UK, Sweden
Rach and Heinze (2017)	Empirical	Quantitative	182 university students (questionnaire, students’ scripts from a test)	Germany
Ufer et al. (2017)	Empirical	Quantitative	323 university students (questionnaire)	Germany
Burazin and Lovric (2018)	Theoretical	–	–	Canada
Hausberger (2018)	Theoretical	–	–	France
Kouvela et al. (2018)	Empirical	Qualitative	10 university students, 42 lecturers (observation of lessons, questionnaire, interview)	UK
Thoma and Nardi (2018)	Empirical	Qualitative	22 university students (students’ scripts from the final examination)	UK
Di Martino and Gregorio (2019)	Empirical	Qualitative	127 university students (questionnaire, interview)	Italy
Dibbs (2019)	Empirical	Qualitative	8 university students (interview)	USA
Koichu and Pinto (2019)	Theoretical	–	–	Israel
Kosiol et al. (2019)	Empirical	Quantitative	120 students (school qualification grade, test results, questionnaire, exam scores)	Germany
Lyakhova and Neate (2019)	Empirical	Quantitative	236 university students (questionnaire, interview, school results)	UK (Wales)
Schüler-Meyer (2019)	Empirical	Qualitative	15 school students in Germany (videotape of the lessons)	The Netherlands
Sommerhoff and Ufer (2019)	Empirical	Quantitative	114 school students, 66 university students, 273 mathematicians (questionnaire)	Germany
Deeken et al. (2020)	Empirical	Qualitative	952 lecturers (questionnaire)	Germany
Gueudet and Thomas (2020)	Theoretical	–	–	France, New Zealand
Rach and Ufer (2020)	Empirical	Quantitative	1553 university students (test results)	Germany
Artigue (2021)	Theoretical	–	–	France
Frank and Thompson (2021)	Empirical	Quantitative	356 university students (students’ production, analysis of precalculus textbooks)	USA
Geisler and Rolka (2021)	Empirical	Quantitative	104 universities students (paper and pencil questionnaire)	Germany
Ghedamsi and Lecorre (2021)	Empirical	Mixed	57 university teachers in Tunisia (questionnaire, observation of lessons, analysis of calculus text)	Tunisia, France
Hochmuth et al. (2021)	Theoretical	–	–	Germany, Canada, UK
O’Shea and Breen (2021)	Empirical	Qualitative	10 university students (semi-structured interviews)	Ireland

The data about the university affiliation of the authors and the geographical contexts where the studies were developed underlines again an issue of representation: the largest number of contributions comes from authors working in European universities reporting studies developed in Europe (63%). Research concerning other regions, such as South America, Asia, Africa, is almost completely absent in the identified corpus.

Concerning the analysis of the methods in the empirical studies, we found a balance between qualitative (43%) and quantitative (37%) approaches (20% used a mixed approach), as well as a variety of instruments and targets (summarised in Table 5).

**Table 5:** Targets and instruments

Target	Instruments and indicators
Students	Prompts in mathematical tasks, questionnaires, biographical and structured interviews, Likert scales, journals, school results, academic results
School teachers	Questionnaires, interviews, direct observations
Lecturers	Questionnaires, interviews, direct observations
Other targets: National School Standards, textbooks	

In particular, the analysis of National School Standards or textbooks allows us to compare different contexts, leading to the awareness that the systems in school and university are culturally embedded (Frank & Thompson, 2021). To this aim, Vollstedt et al. (2014) elaborated a framework for analysing and comparing mathematics textbooks.

The analysis of the research methods highlights two related issues: most data are collected through online surveys (but the work by Geisler and Rolka (2021) represents a recent exception) and the sample is almost exclusively a sample of volunteers. This latter aspect, recurrent in the mathematics education research, appears to be particularly relevant in STT research since it involves adults, asking them to report an event often perceived as a personal failure.

### Theoretical models

The 55 selected papers include a great variety of theoretical frameworks: several of these theoretical approaches are related to Tall’s ideas of advanced mathematical thinking and the three worlds of mathematics (e.g., Deeken et al., 2020; Hong et al., 2009), others are based on the Anthropological Theory of the Didactic (e.g., Hausberger, 2018), and others are within the theory of commognition by Sfard (2008). In the latter case, the focus is on the shift of mathematical discourses between secondary school and university (Thoma & Nardi, 2018).

Recently, more authors have adopted frameworks related to affective constructs not originally developed in the field of STT: Hernandez-Martinez et al. (2011) focused on mathematical identity adopting the perspective of Sfard and Prusak (2005); Ufer et al. (2017) conceptualised interest within the person-object theory of interest by Krapp (2002); Dibbs (2019) used engagement theory (Fredricks et al., 2004) to study students’ affective reactions to failure in a calculus course; Geisler (2021) analysed the role of attitude in the dropout from university mathematics within the three-dimensional model of attitude (Di Martino & Zan, 2010).

In 2008, Clark and Lovric observed that ‘perhaps the most notable feature of the existing body of research on transition is the absence of a theoretical model’ (p. 25). In a special issue of the *Mathematics Education Research Journal* dedicated to transitions, two specific (and influential) models were discussed: the three worlds of mathematics by Tall (2008) and the rite of passage by Clark and Lovric (2008).

The three worlds of Mathematics is a theory about the development of mathematical thinking that Tall presented at PME in Bergen (Tall, 2004). In this theory, the development of mathematical thinking is described as the development of perceptions of three different but interrelated worlds. In 2008, Tall used this theory to focus on the changes in thinking involved in the STT and on the individual development needed for this transition. According to this theoretical model, there are three fundamental mental structures that shape long-term learning and mathematical thinking: recognition of patterns, repetition of sequences of actions, and language. In this cognitive and epistemological perspective, Tall (2008) identified ‘three worlds of mathematics that develop in sophistication in quite different ways’ (p. 7): conceptual embodiment, proceptual symbolism (APOS theory is included in this world), and axiomatic formalism. Tall explained how, in his view, the blending of embodiment and symbolism gives a more accurate way of developing sophistication in mathematical thinking. In the final version of his theoretical model, Tall (2013) included an affective dimension recognising the role of emotions in the interpretation of previous experiences with mathematics, and therefore in the individual development of mathematical thinking.

**Fig. 2:**
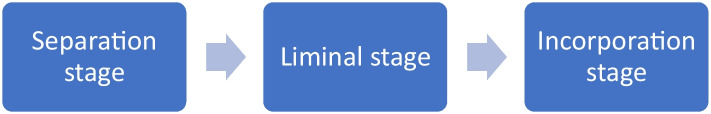
The three stages of the rite of passage

Clark and Lovric (2008) adapted a well-established anthropological theory to the study of STT: that of rites of passage. This model recognised three stages in STT (Fig. 2).

The liminal stage includes the period from the last part of high school to the first part of university. It is characterised by an unavoidable crisis, known mathematical routines are challenged, and first year students need to find their place in a new mathematics community. This model appeared initially strongly influenced by the cognitive perspective in STT; Clark and Lovric (2008) discussed only the cognitive shock of the passage from informal to formal language and reasoning in mathematics. A year later, Clark and Lovric (2009) recognised that the rite of passage inevitably leads to the emergence of affective reactions: the initial reaction of euphoria, the feelings of inadequacy during the crisis, and the recovery after the resolution of the crisis. These affective reactions are particularly strong for those students who were successful in secondary school: this is the case of most of the first-year students in mathematics (Di Martino & Gregorio, 2019).

The rite of passage model has two main implications. First, dealing with a significant crisis is a necessary step for a successful passage: instead of avoiding the crisis — for example making the new context like the old one — the crisis needs to be understood to offer students support to overcome it. As Thomas and Klymchuk (2012) observed: ‘the existence of demanding aspects of transition that are difficult to control is not in itself a good enough reason to ignore those that can be managed to produce a better experience for students’ (p. 285). An unsuccessful rite of passage may result in a never completed incorporation stage, and students’ dropout can be interpreted in this perspective. Second, the rite of passage model stresses the fact that socio-cultural aspects cannot be disregarded in the analysis of STT: the old and new communities through which the rite of passage is accomplished are context specific.

### Main themes of research

Through the coding process, we identified, inductively, four main research themes. We describe them in turn below.

1. The mathematical gap between secondary school and university. This gap is described in terms of students’ thinking (Godfrey & Thomas, 2008), approach and content (Brandell et al., 2008), acceptance criteria for justification (Selden, 2012; Sommerhoff & Ufer, 2019) and for legitimate mathematical activity (Jablonka et al., 2017), teaching style and assessment (Thomas & Klymchuk, 2012), didactic contract (Pepin, 2014b) and messages students receive (Kouvela et al., 2018), identity (Jooganah & Williams, 2016), and high school calculus outcomes and university calculus requirements (Ghedamsi & Lecorre, 2021). These studies are mainly conducted within a socio-cultural perspective, adopting the three worlds of mathematics framework (Tall, 2008). In recent years, the approach to STT has become more holistic, including new viewpoints. For example, some studies consider the social and discursive perspective related to the analysis of commognitive conflicts (Thoma & Nardi, 2018); others describe the gap between school and university mathematical experiences through the description of the perception of the main actors involved: students (e.g., Hernandez-Martinez et al., 2011; O’Shea and Breen, 2021), schoolteachers (Hong et al., 2009), lecturers (Klymchuk et al., 2011; Deeken et al., 2020), or compare these different perceptions (Corriveau, 2017).
2. The potential of technology to facilitate the STT. Two issues emerge within this category: the effective use of CAS (Computer Algebra Systems) in STT (Hong & Thomas, 2015; Varsavsky, 2012) and the analysis of the potential of ICT (Information and Communication Technology) to facilitate the STT (Bardelle & Di Martino, 2012; Daza et al., 2013). It is interesting to observe that these kinds of studies are limited to the period 2012–2015: the recent events related to the pandemic could (should?) generate new interest towards this line of research in STT (Chan et al., 2021).
3. The factors correlated with academic success. The studies in this category consider a wide range of cognitive, social, and affective factors and are mostly quantitative, involving large samples. Their explicit goal is to highlight significant correlations between academic success and other factors, such as students’ attitudes (Geisler, 2021), standardised test results (Culpepper et al., 2010), attended secondary school (Adamuti-Trache et al., 2013), prior knowledge (Rach & Ufer, 2020), students’ learning prerequisites (Rach & Heinze, 2017), interest (Kosiol et al., 2019), and students’ beliefs (Geisler & Rolka, 2021).
4. Failure in STT. This category represents studies of failure to transition: they are mostly qualitative, based on the collection of narratives, often framed as case studies (Hernandez-Martinez, 2016). The shared assumption of these studies is that the difficulties in STT are inevitable — according to Clark and Lovric (2009) even essential — and there is a thin line between success and failure. The description and interpretation of the failure is considered a significant key to understand, prevent, and overcome the students’ difficulties in STT. The definition of failure in STT varies in these studies: there is a *local* meaning, i.e., negative results in some first-year university exam (Dibbs, 2019) and a *global* meaning, i.e., students who leave university studies (Di Martino & Gregorio, 2019).

These four themes of research are highly specific to mathematics. This is evident for the first two themes which deal with subject-related issues. However, it is also the case for the latter two themes. Regarding the third theme, the factors considered are mathematics-related: for example, students’ beliefs and attitudes reveal students’ perceptions about the nature of the mathematics they are encountering at university. The fourth theme, that of failure/success in STT, is related to the students’ vision of the nature of mathematics. A change in one’s mathematical theory of failure/success is a change in one’s vision of mathematics. The mathematics epistemology conveyed by the mathematical culture in the new university environment affects students’ mathematical theories of success through the introduction of new mathematical symbols, knowledge, customs, and requirements for success. Therefore, all the elements of difficulty, not only the cognitive ones, but also the affective and sociocultural ones, are highly specific to mathematics.

## Conclusion and directions for research

First, a meta-reflection about literature searches based on fully automated search engines such as Google Scholar. Such searches not only present an issue of non-reproducibility (Gusenbauer & Haddaway, 2020) but also present an issue of control (or rather of poor control). These searches are affected by external and contextual factors. The list produced is based on an unknown ranking algorithm (Beel & Gipp, 2009) and the profiling process and geolocation of who develops the search also play a role. The introduction of external selection criteria not fully controlled by researchers is a significant dilemma for the scientific community.

Notwithstanding this significant issue, the academic search engines are an essential resource to develop systematic literature reviews. We believe that adopting the recommendations of Haddaway et al. (2015) — for example using a primary and a supplementary search engine, adopting a suitable search query, and considering a large sample of the papers — it is possible to obtain a representative picture of the state of the art in the field investigated, also highlighting the so called ‘grey literature’ (Haddaway et al., 2015).

Our review of the research literature on STT from 2008 to 2021 highlights the variety of theoretical frameworks in use, the growing awareness of the complexity of phenomena at play in STT and the consequent adoption of a more holistic approach to STT that goes beyond a purely cognitive interpretation, including socio-cultural and affective issues. The first studies focused on the cognitive and epistemological obstacles of the shift toward advanced mathematical thinking have been integrated and complemented by studies considering social, cultural, and affective issues in the last decade. On the other hand, Artigue (2021) recently underlined how ‘the socio-cultural turn... has not yet impacted research’ (p. 9) at university level and we argue that the ‘affective turn’ has also not been completely fulfilled in STT research.

The research results obtained considering social, cultural, and affective issues have already some clear implications for the teaching and learning of mathematics at secondary school and at university level. Since the seminal work of Tall (1991), we know much about the difference between tertiary mathematics and secondary school mathematics: mathematics is understood and presented in a different (advanced) way at the university level. The more recent research on the STT highlights how tertiary transition involves other changes than the purely cognitive and other actors. Many difficulties the learners experience in the passage from school mathematics to university mathematics appear to be related to a sudden change in their mathematics identity (Hernandez-Martinez et al., 2011). Many successful students develop a different view of mathematics in their passage to university, often perceiving that their ability in mathematics is suddenly reduced and, consequently, developing very strong negative emotions in their university experience (Geisler & Rolka, 2021). This affective phenomenon is however related to an epistemological aspect: the meaning of being a successful student in mathematics. The individuals’ theory of success in mathematics are rooted and consolidated during the school experience (Di Martino & Zan, 2010), going often into crisis during the tertiary transition. Whether the development of the students’ theories of success during STT is unavoidable and related to the epistemology of the advanced mathematics encountered at university is an open question at the boundary between epistemological and didactical issues.

Concerning the second research question we posed, we identified four main themes of the STT research: the mathematical gap between secondary school and university, the potential of technology to facilitate STT, and the factors correlated to academic success and to academic failure in STT. These strands of research produced significant outcomes. However, we believe that more research is needed to improve our understanding of the relationship between cognitive, affective, social, and cultural aspects of the STT. We encourage studies that consider the dynamic nature of STT — that consider STT as a process — and bring into play the cultural context in which this crisis takes place. In this perspective, comparative studies between cultural or institutional contexts are still rare: an interesting exception is represented by the study of Deeken et al. (2020) that, through the Delphi method, described which students’ mathematical abilities are considered minimal prerequisites by university mathematics lecturers. Studies involving and comparing several institutions or countries are rare (Di Martino et al., 2022, is one such example), but much needed, since they can help to understand the role of contextual factors and the generalisability of studies conducted in a specific context. Further studies in this direction would surely represent a valuable addition to the current body of research.

From a methodological point of view, the analysis of existing research highlights on the one hand the use of several different instruments, approaches, and samples. On the other hand, it points to two significant issues. First, most data are collected through online surveys: this choice is ‘not neutral’. Psychologist have discussed the impact of computer versus paper–pencil survey in collecting self-reports (Bates & Cox, 2008) and, in mathematics education, recent publications address the impact in students’ performance in online versus pen and paper tasks (Lemmo, 2021).

Second, the participants of these studies are almost exclusively volunteers, and we need to consider and discuss the limitations related to this. The effects of a sample of volunteers appear to be particularly significant in the analysis of failure in STT, where students who drop out or are about to do so are often the focus of research. The volunteer sampling in the STT may create a bias towards higher achieving students (Vollstedt et al., 2014).

In our overview, we included papers such as Doukhan (2020), Griese (2017), Jablonka et al. (2017), and Hong and Thomas (2015). Those papers focus on STT concerning non-specialist students: i.e., students who study mathematics as part of their degree but are not enrolled in a degree course in mathematics. The study of the differences in the mathematical transition to the various STEM degrees — the differences in the mathematical transition between specialist and non-specialist students — appears to be a significant perspective for further research.

These differences can be related to the different mathematical identities of the first-year students, to the different degrees of discontinuity of mathematical contents (for example, calculus) as they are presented at school and as they are presented in the different degrees, as well as to the different mathematical practices and requests in the different degrees. In our view, this research should also involve epistemological, socio-cultural, and affective aspects.

Finally, the result of our systematic review confirms a need of our community: that of learning more about STT in the areas of the world that are not represented in our final corpus. This is not only an important issue of equity and participation, but — considering the role of cultural, affective, and social factors in the STT — our limited knowledge of many educational contexts signifies a limited understanding of the STT, an understanding too culturally bound. We firmly believe that the mathematics education community should make all possible efforts to support researchers from underrepresented educational contexts interested in developing research about STT.

## Data Availability

The datasets analysed during the current study are available from the correspondent author upon reasonable request. These datasets were derived from the following public domain resources: Google Scholar and ERIC.

## Footnotes

1 For the theoretical studies, we reported and considered the author’s nationality.

2 The PME conference was not held in 2020, due to the Covid-19 pandemic.
